# Matrix Rigidity‐Dependent Regulation of Ca^2+^ at Plasma Membrane Microdomains by FAK Visualized by Fluorescence Resonance Energy Transfer

**DOI:** 10.1002/advs.201801290

**Published:** 2018-12-18

**Authors:** Tae‐Jin Kim, Lei Lei, Jihye Seong, Jung‐Soo Suh, Yoon‐Kwan Jang, Sang Hoon Jung, Jie Sun, Deok‐Ho Kim, Yingxiao Wang

**Affiliations:** ^1^ Neuroscience Program and the Beckman Institute for Advanced Science and Technology University of Illinois at Urbana‐Champaign Urbana IL 61801 USA; ^2^ Department of Bioengineering and Institute of Stem Cell and Regenerative Medicine University of Washington Seattle WA 98195 USA; ^3^ Department of Biological Sciences Integrated Biological Science and Institute of Systems Biology Pusan National University Pusan 46241 Republic of Korea; ^4^ Department of Bioengineering Institute of Engineering in Medicine University of California at San Diego La Jolla CA 92093 USA; ^5^ Convergence Research Center for Diagnosis Treatment Care of Dementia Korea Institute of Science and Technology (KIST) Seoul 02792 Republic of Korea; ^6^ Department of Integrated Biological Science Pusan National University Pusan 46241 Republic of Korea; ^7^ Natural Products Research Center Korea Institute of Science and Technology (KIST) Gangneung 25451 Republic of Korea; ^8^ Department of Cell Biology and Bone Marrow Transplantation Center of the First Affiliated Hospital Zhejiang University School of Medicine Hangzhou 310058 China; ^9^ Institute of Hematology Zhejiang University and Zhejiang Engineering Laboratory for Stem Cell and Immunotherapy Hangzhou 310058 China; ^10^ Department of Bioengineering University of Illinois at Urbana‐Champaign Urbana IL 61801 USA

**Keywords:** biosensors, calcium, focal adhesion kinase, live cell imaging, matrix rigidity

## Abstract

The dynamic regulation of signal transduction at plasma membrane microdomains remains poorly understood due to limitations in current experimental approaches. Genetically encoded biosensors based on fluorescent resonance energy transfer (FRET) can provide high spatiotemporal resolution for imaging cell signaling networks. Here, distinctive regulation of focal adhesion kinase (FAK) and Ca^2+^ signals are visualized at different membrane microdomains by FRET using membrane‐targeting biosensors. It is shown that rigidity‐dependent FAK and Ca^2+^ signals in human mesenchymal stem cells (hMSCs) are selectively activated at detergent‐resistant membrane (DRM or rafts) microdomains during the cell–matrix adhesion process, with minimal activities at non‐DRM domains. The rigidity‐dependent Ca^2+^ signal at the DRM microdomains is downregulated by either FAK inhibition or lipid raft disruption, suggesting that FAK and lipid raft integrity mediate the in situ Ca^2+^ activation. It is further revealed that transient receptor potential subfamily M7 (TRPM7) participates in the mobilization of Ca^2+^ signals within DRM regions. Thus, the findings provide insights into the underlying mechanisms that regulate Ca^2+^ and FAK signals in hMSCs under different mechanical microenvironments.

## 1 Introduction

Cell‐based therapeutics are revolutionizing the medicine field.^1^ One promising branch is stem cell‐based therapy, which has developed from preclinical to early clinical studies for treatment of various diseases.^2^ Human mesenchymal stem cells (hMSCs) are a type of adult stem cells (ASCs) that are multipotent, easily accessible, and can be expanded ex vivo, providing great potential for clinical applications.^3^ However, insufficient stem cell adhesion and survival in vivo remains a problem, even though it can be partly addressed by tissue engineering and ex vivo genetic modifications.^4^ Indeed, the mechanical environment of cells, including factors such as substrate stiffness, has been shown to influence adhesion,^5^ which is essential for hMSC survival, proliferation, and differentiation.^6^ As such, the cell adhesion process not only links the extracellular matrix (ECM) and cytoskeleton chemically, but also establishes mechanical coupling between the ECM and the cell.^7^ Therefore, understanding the molecular mechanism of hMSC adhesion, especially in the context of its mechanical environment, is necessary for the development of scaffold and active biological materials to enhance cell adhesion for hMSC‐based therapy.

Cell adhesion to the surrounding matrix starts with the binding of integrin receptors in the plasma membrane to ECM proteins. Binding to matrix proteins such as fibronectin and collagen leads to integrin clustering and subsequent downstream assembly of both mechanical structures and chemical signaling complexes, including adaptor proteins, cytoskeletal components, catalytic signaling proteins, and secondary messengers.^8^ Focal adhesion kinase (FAK) is a key component of integrin‐mediated signal transduction at focal adhesion complexes, which consequently mediates cell adhesion and migration.^9^ FAK has been extensively studied during cell–matrix adhesion process on glass, but relatively less investigated when cells are adhered on softer substrates than glass.^10^ Integrins also induce intracellular Ca^2+^ increase in various cell types.^11^ This Ca^2+^ signal then directly and/or indirectly regulates adhesion through Ca^2+^‐dependent proteins such as myosin II and calpain.^12^, ^13^ Most studies designed to evaluate cell adhesion utilize cell types other than hMSCs. Our previous studies revealed that hMSCs are highly sensitive to the mechanical microenvironment, displaying spontaneous Ca^2+^ oscillations which is dependent on the cell adhesion on mechanical environment.^14^ However, there is little understanding on the regulation of FAK and Ca^2+^ during the cell–matrix adhesion process of hMSCs under different mechanical microenvironment. Even less is known about the spatial organization of activation patterns of FAK and Ca^2+^ at the plasma membrane, which is structurally organized into two functional microdomains called detergent‐resistant membrane (DRM) and non‐DRM regions.^15^, ^16^ This spatial organization of FAK and Ca^2+^ is crucial for the initiation of FAK and Ca^2+^ signals.

The DRM, known as lipid rafts membrane, is enriched with sphingolipids and cholesterol, whereas non‐DRM domains lack these lipid compositions.^17^ Lipid rafts are not only enriched with resident integral membrane proteins such as caveolin and flotillin, they are also connected to extracellular proteins through glycophosphosphatidylinositol (GPI) anchors containing long chain fatty acids.^16^, ^18^ Cytoplasmic proteins can be integrated into lipid rafts by the modification of a dual myristoylation/palmitoylation motif or palmitoylation on cysteine residues.^19^, ^20^ Such distinct characteristics of membrane microdomains can contribute to the specific dynamics of cellular signaling and their physiochemical regulation owing to the differential distribution of membrane‐associated proteins at plasma membrane microdomains. However, it remains unclear how signaling events are compartmentalized by specialized microdomains due to limitations in available methodologies.

In this study, we take advantage of membrane‐targeting fluorescent resonance energy transfer (FRET)‐based FAK and Ca^2+^ biosensors to investigate the regulation of these two signals during cell–matrix adhesion process in hMSCs seeded on substrates with different stiffness. Through live single cell imaging, we first demonstrate that rigidity‐dependent FAK and Ca^2+^ signals are selectively enriched at detergent‐resistant membrane (DRM) microdomains in a concerted manner, but not at non‐DRM during this adhesion process. Furthermore, we report that transient receptor potential subfamily M7 (TRPM7) is involved in the regulation of matrix rigidity‐dependent Ca^2+^ signals at DRM microdomains, which is mediated by the functional FAK and the integrity of lipid rafts.

## 2 Results and Discussion

### 2.1 FAK Activation at DRM Microdomains Is Mediated by Matrix Rigidity

**Figure 1:**
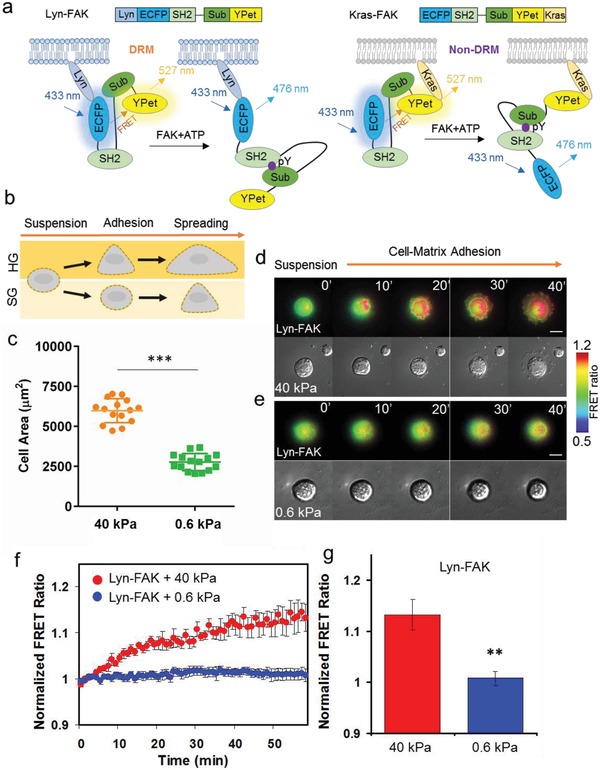
Matrix rigidity‐dependent focal adhesion kinase (FAK) activation at DRM microdomains during the adhesion process. a) Schematic drawings of the underlying mechanism illustrating the conformational changes in FAK biosensors enabling detection of FAK signals at two different plasma membrane microdomains, DRM (Lyn‐FAK) and non‐DRM (Kras‐FAK). FAK phosphorylation of the substrate enables its interaction with the SH2 domain, leading to a conformational change in the FAK biosensor. Lyn‐FAK and Kras‐FAK biosensors can be tethered at DRM and non‐DRM microdomains, respectively. b) Schematic drawings of adhesive hMSCs exposed to either hard (HG, young's modulus, 40 kPa) or soft gels (SG, 0.6 kPa). c) Analysis of cell area 40 min after seeding on substrates with different rigidities (*n* = 15, ***P < 0.001). d,e) Time‐lapse FRET images of Lyn‐FAK in hMSC grown on 40 and 0.6 kPa during the cell–matrix adhesion process. Hot and cold colors indicate high and low FAK activities, respectively. Scale bar = 20 µm. f,g) Dynamic and average changes in the FRET ratio of Lyn‐FAK biosensor in hMSCs cultured on 40 and 0.6 kPa gels. All error bars are s.e.m (*n* = 7–10, **P < 0.01).

It is known that matrix rigidity has a significant impact on cell adhesion and spreading via integrin‐cytoskeleton linkages.^21^ FAK plays a crucial role in the regulation of integrin‐cytoskeleton networks.^22^ However, it remains a challenge to elucidate whether/how FAK activity is spatiotemporally controlled at the plasma membrane microdomains in the early stages of cell adhesion and spreading processes. To unravel this, we developed and utilized two kinds of membrane‐targeting FAK biosensors based on FRET technology. These biosensors were designed to detect changes in FAK activity within specific membrane microdomains. As illustrated in **Figure** **1**a, interaction between the SH2 domain of the biosensor and the substrate phosphorylated by FAK triggers a conformational change in the biosensor. This conformational change leads to alteration of the distance/orientation between the enhanced cyan fluorescent protein (ECFP) and the yellow fluorescent protein variant YPet (yellow fluorescent protein for energy transfer) with a FRET ratio change. Quantification of this change provides an index to measure levels of FAK‐induced phosphorylation (Figure 1a). A DRM‐targeting FAK biosensor, called Lyn‐FAK, was then engineered containing a lipid raft‐targeting motif (MGCIKSKRKDNLNDDE) originated from Lyn kinase to the N‐terminus of cytosolic FAK, which enables the tethering of this sensor to the DRM microdomain. In contrast, a non‐DRM‐targeting FAK biosensor, called Kras‐FAK, holds a non‐raft‐targeting motif, a prenylation substrate sequence from Kras (KKKKKKSKTKCVIM) to the C‐terminus of the sensor.^23^ These biosensors were separately transfected into hMSCs, and the cells were then seeded on polyacrylamide (PA) hydrogels with a Young's modulus of 0.6 or 40 kPa prior to imaging experiments and analysis (Figure 1b). We first showed that hMSCs seeded on 40 kPa gel displayed larger surface areas indicating a better spreading compared to cells on 0.6 kPa gel (*n* = 15, ***P < 0.001) (Figure 1c). Applying FRET biosensors, we further observed that the FRET ratio of Lyn‐FAK at DRM microdomains increased by 15% from the basal level within 1 h upon adhesion on 40 kPa gel surface (*n* = 7), but not on 0.6 kPa gel (*n* = 10) (Figure 1e–g). These results suggest that the FAK activity within DRM microdomains can be induced upon cell adhesion, which is dependent on matrix rigidity. FAK has been shown to participate in mechanosensing.^24^ However, it was not known whether FAK is exclusively accumulated or recruited to adhesive partners at DRM microdomains during cell–matrix adhesion process. While previous studies have shown chemically mediated FAK recruitment to DRM microdomains, our findings provide the first evidence that such recruitment can also be mediated by mechanical factors such as matrix rigidity.

### 2.2 Distinct FAK Activation at Membrane Microdomains

**Figure 2:**
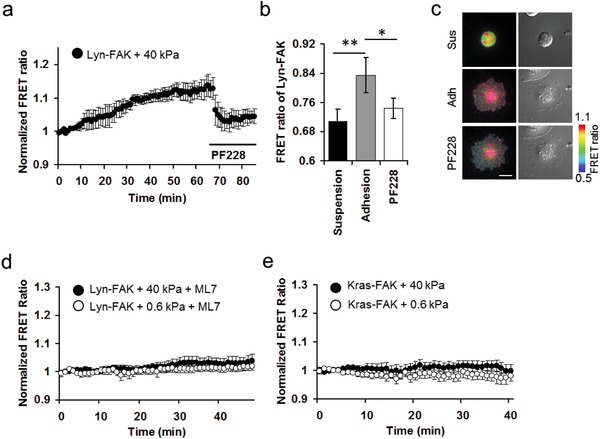
Distinct FAK activations at different plasma membrane microdomains in response to matrix rigidity. a–c) Time‐lapse analysis of Lyn‐FAK signal during the cell–matrix adhesion process and treatment with PF228, a specific inhibitor of FAK. Bar graphs represent the FRET ratio in suspension, adhesion/spreading in the presence and absence of PF228 (*n* = 3, *P < 0.05, and **P < 0.01), Scale bar = 20 µm. d) The FRET ratio of Lyn‐FAK in response to 40 or 0.6 kPa in the presence of ML‐7, an inhibitor of MLCK (*n* = 5–7). e) The FRET ratio of Kras‐FAK biosensor reflecting the FAK signal at non‐DRM in hMSC cultured on 40 or 0.6 kPa gels (*n* = 9) in the adhesion process.

To confirm whether the obtained FRET signals originated from FAK activity, we treated hMSCs with a specific FAK inhibitor, PF228, as a control to suppress the upregulated FAK activity. When the cells were in suspension, the FRET ratio of Lyn‐FAK was relatively lower than those in adhesion (*n* = 3, *P < 0.05, and **P < 0.01) (**Figure** **2**a–c). Immediately after PF228 treatment, rigidity‐dependent Lyn‐FAK signals were attenuated, suggesting that the observed FAK activation was specific (Figure 2b,c) and occurred predominantly (Figure 2e) at DRM microdomains. Since myosin light chain kinase (MLCK) is known to regulate actomyosin contractility and mechanical support of the cells, which are crucial for the structural integrity of DRM microdomains,^13^ we further examined whether ML‐7, an inhibitor of MLCK, regulates FAK activity at DRM microdomains. Our results clearly show that ML‐7 pretreatment markedly inhibited the FAK activity at DRM microdomains even when hMSCs were seeded on rigid matrix (*n* = 5–7) (Figure 2d), suggesting the involvement of MLCK and its associated actomyosin contractility and structural support in regulating DRM FAK activities. Our previous results suggest that FAK activities are mostly concentrated in DRM regions in HT1080 cancer cells.^23^ Consistently, no significant increase in Kras‐FAK signal was detected at non‐DRM microdomains during the cell–matrix adhesion process in hMSCs (*n* = 9) (Figure 2e).

### 2.3 Ca^2+^ Mobilization at the Plasma Membrane Microdomains

**Figure 3:**
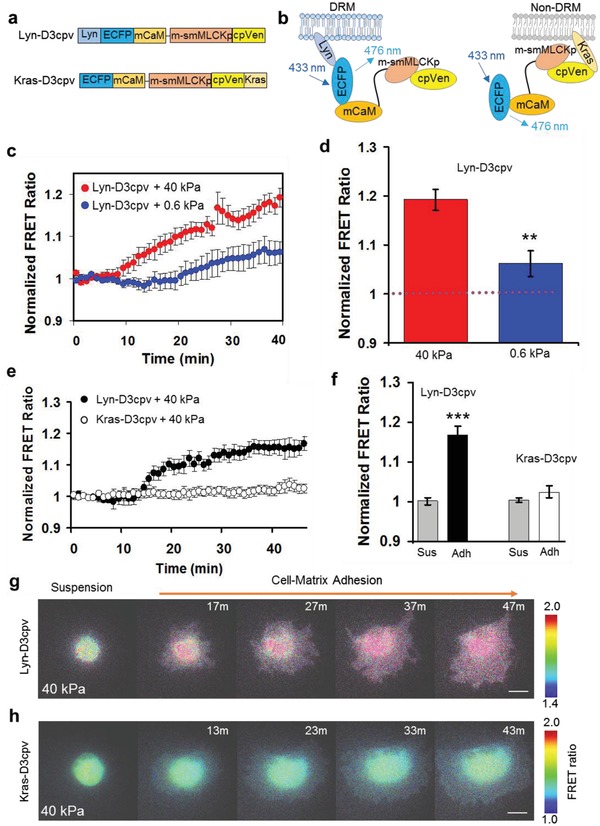
Matrix rigidity‐dependent Ca^2+^ activation at membrane microdomains during the adhesion process. a,b) Schematic drawings of the underlying mechanism by which Ca^2+^ biosensors target DRM (Lyn‐D3cpv) and non‐DRM regions (Kras‐D3cpv). Ca^2+^ binding to mutated calmodulin (mCaM), which consequently interacts with the intramolecular m‐smMLCKp (smooth muscle myosin light chain kinase peptide), results in a conformational change in the Ca^2+^ biosensors. c,d) The FRET ratio indicates a Ca^2+^ signaling activity at DRM (Lyn‐D3cpv) in hMSCs cultured on 40 and 0.6 kPa gels (*n* = 5–8, **P < 0.01, ***P < 0.001). Bar graphs indicate the quantitative analysis of Ca^2+^ signaling activity 40 min after cell seeding. e,f) The FRET ratio showing the Ca^2+^ signaling activity at DRM (Lyn‐D3cpv) and non‐DRM (Kras‐D3cpv) in hMSC cultured on 40 kPa. Sus; Suspension, Adh; Adhesion. (*n* = 7–8, ***P < 0.001). g,h) Time‐lapse FRET images of Lyn‐D3cpv and Kras‐D3cpv in hMSC during the cell–matrix adhesion process. The hot and cold colors represent high and low FRET ratios, indicating high and low Ca^2+^ signaling activities, respectively. Scale bar = 20 µm.

Similar to FAK, Ca^2+^ signals also play an important role in cell adhesion and spreading.^13^ Accordingly, we examined how Ca^2+^ signals could be activated at plasma membrane microdomains. To perform this study, two distinct types of FRET‐based Ca^2+^ biosensor were engineered to tether at DRM and non‐DRM microdomains using Lyn and Kras peptide sequences, respectively (**Figure** **3**a,b). These membrane‐targeting Ca^2+^ biosensors, Lyn‐D3cpv and Kras‐D3cpv, can detect the FRET changes caused by the change of Ca^2+^ concentration at DRM and non‐DRM microdomains. These Ca^2+^ sensors showed similar levels of FRET increase in response to treatment with ionomycin, an ionophore that increases the plasma membrane permeability and raises the intracellular level of Ca^2+^ (Figure S1, Supporting Information), suggesting that similar Ca^2+^ influx occurs at these two different membrane microdomains DRM versus non‐DRM regions with ionomycin treatment. Interestingly, the Ca^2+^ increase during cell–matrix adhesion process selectively occurred only at the DRM microdomains in a rigidity‐dependent manner, but with undetectable signals at non‐DRM regions (Figure 3c,d, *n* = 5–8, **P < 0.01, ***P < 0.001, Figure 3e–h, *n* = 7–8, ***P < 0.001 and Figure S2, Supporting Information). This pattern of calcium regulation during adhesion process is similar to that of FAK activation.

We have previously reported that the membrane targeting motifs should not affect the function of fused FRET biosensors.^23^, ^25^ Our findings hence offer the first evidence that Ca^2+^ signals are differentially mobilized at different plasma membrane microdomains during the process of cell–matrix adhesion in hMSCs. It is possible that there is a high level of buffer proteins such as calmodulin specifically localized at the non‐raft regions, which can bind to and neutralize free Ca^2+^ diffused from other subcellular domains, for example, DRM and cytosolic organelles. This is consistent with earlier reports that there are different compositions of membrane proteins at different microdomains during cell adhesion processes, including Ca^2+^ channels, integrins, and FAK.^26^, ^27^

### 2.4 The Regulation of Ca^2+^ Mobilization at DRM Microdomain by FAK

**Figure 4:**
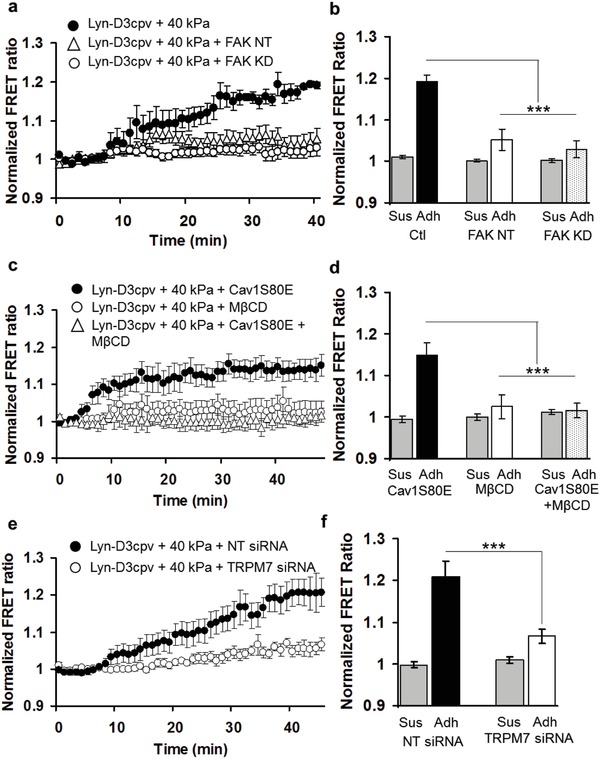
Regulation of Ca^2+^ signals and its associated molecules at DRM by FAK during the cell–matrix adhesion process. a,b) Ca^2+^ mobilization and its quantitative analysis at DRM in hMSCs in response to 40 kPa in the absence or presence of FAK mutants (FAK NT and KD) (Ctl, *n* = 3; FAK NT, *n* = 7; FAK KD, *n* = 8, ***P < 0.001). c,d) Effect of Caveolin‐1 mutation (Cav1S80E) and cholesterol depletion on Ca^2+^ signaling activity. Cholesterol depletion and hence lipid raft disruption by MβCD, but not Cav1S80E, at DRM inhibits Ca^2+^ mobilization during adhesion process in hMSCs cultured on 40 kPa (*n* = 5–7, ***P < 0.001). e,f) Calcium mobilization during the adhesion process on 40 kPa in hMSCs cotransfected with Lyn‐D3cpv and TRPM7 siRNA or control siRNA (*n* = 5–9, ***P < 0.001).

In this study, we found that within DRM microdomains, hMSCs displayed higher Ca^2+^ mobilization and FAK activation during the adhesion process, both of which are dependent on the magnitude of matrix rigidity. In contrast, these two signals were poorly activated at non‐DRM regions regardless of matrix rigidity. To further examine whether Ca^2+^ signals are correlated with FAK signals, we took advantage of two FAK mutants, FAK NT (a negative mutant, N‐terminal tail of FAK) and FAK KD (a kinase dead mutant). These mutants were cotransfected into cells expressing the calcium biosensor Lyn‐D3cpv. As shown in **Figure** **4**a,b and Figure S3 (Supporting Information), FAK NT and FAK KD caused reduced Ca^2+^ signaling activity at DRM microdomains (Ctl, *n* = 3; FAK NT, *n* = 7; FAK KD, *n* = 8, ***P < 0.001). Pharmacological inhibitor PF228 or ML‐7 also decreased Ca^2+^ mobilization within the DRM region (Figure S4, Supporting Information). These results indicate that Ca^2+^ mobilization at the DRM region is regulated by FAK during the cell–matrix adhesion process.

The physiological interpretation of these phenomena requires further investigation to understand detailed mechanisms with regard to how FAK regulates Ca^2+^ mobilization. One possibility is that FAK directly affects Ca^2+^ channels at DRM regions. Even though FAK is mostly interacting with integrins at the plasma membrane, it is possible that FAK can physically interact with mechanosensitive membrane channels.^28^ Another possibility could be that FAK affects Ca^2+^ channels indirectly in an integrin‐dependent manner. In fact, FAK modulates different types of voltage‐gated Ca^2+^ channels at the plasma membrane via integrins.^29^ Ca^2+^ influx through the plasma membrane has also been shown to closely correlate with the activation of high affinity β2 integrin and subsequent adhesive signals due to their dynamically coupled events.^30^

### 2.5 Depletion of Cholesterol by MβCD Inhibits Ca^2+^ Mobilization at DRM Region, but Not by Caveolin‐1

It is reported that cholesterol, along with caveolin, plays an important role in signal transduction at DRM microdomains.^31^ In fact, cholesterol depletion by MβCD appears to cause the downregulation of FAK.^32^ It is possible that caveolin‐1, a key protein of caveolae, interacts with integrin β1 and promotes its localization at the DRM region, thereby affecting FAK activity.^33^ Our previous study showed that the platelet derived growth factor (PDGF) induced FAK activation was inhibited by MβCD treatment.^23^ Thus, we examined the role of DRM integrity in regulating the rigidity‐dependent Ca^2+^ mobilization at these local regions during the cell–matrix adhesion process, by depleting cholesterol with MβCD. As shown in Figure 4c,d (*n* = 5–7, ***P < 0.001), we found that MβCD treatment caused significant inhibition of FRET changes in Lyn‐D3cpv calcium biosensor, while a mutant of caveolin‐1 (Cav1S80E) did not affect the Ca^2+^ mobilization at DRM microdomains (Figure S5, Supporting Information). These results suggest that cholesterol at DRM microdomains is crucial in Ca^2+^ mobilization during cell–matrix adhesion process, which is not dependent on the caveolin‐1 function.

Although it is known that caveolae disruption prevents Ca^2+^ influx under mechanical stretch,^34^ it may not have a significant impact on the adhesion process. Alternatively, caveolin may target other TRP channels, for example, TRPC1, instead of TRPM7,^35^ in affecting the stretch‐induced Ca^2+^ influx. While our previous study has shown that cholesterol disruption by MβCD treatment inhibited FAK activation at DRM region upon PDGF stimulation,^23^ caveolin‐1 only specifically links the integrin α subunit, but not β subunit where FAK is coupled, and thus has possibly less effect on FAK activity during the adhesion process.^36^

### 2.6 TRPM7 Contributes to Ca^2+^ Mobilization at DRM Region

The distribution of TRP Ca^2+^ channels at DRM regions is different from that of non‐DRM regions. For example, TRPC3 and TRPC6 are dominantly expressed at non‐DRM regions, but other TRPC channels are expressed throughout the plasma membrane.^27^ TRPM7 is a mechanosensitive Ca^2+^ permeable channel, which is known to dominantly accumulate at DRM microdomains, but not at non‐DRM regions.^37^ Consistently, it was reported that TRPM7 serves as an adhesion‐associated channel that regulates actomyosin contractility.^38^ This suggests that the integrin‐FAK complex might be connected with TRPM7 at DRM microdomain via cytoskeleton (CSK)‐actomyosin network to regulate Ca^2+^ signals. Indeed, previous studies support the note that TRPM7 and integrin‐FAK complex may be closely interrelated. For example, TRPM7 regulates focal adhesions by controlling m‐calpain,^39^ and the inhibition of TRPM7 disrupts the actin cytoskeleton, and the focal assembly of myosin IIA and vinculin, a focal adhesion protein.^40^ We hence examined whether TRPM7 could be involved in Ca^2+^ mobilization at DRM microdomains during the cell–matrix adhesion process. In our previous reports, we have shown that the delivery of TRPM7 siRNA clearly suppressed the expression of TRPM7 in hMSCs.^41^ Using this siRNA method, we observed that TRPM7 knockdown significantly inhibited the rigidity‐dependent Ca^2+^ mobilization at the DRM region, with nontargeting (NT) siRNA (control group) having minimal effects (*n* = 5–9, ***P < 0.001) (Figure 4e,f and Figure S6, Supporting Information). Interestingly, TRPM7 activation by small molecule naltriben, a specific activator of TRPM7 rescued the lack of Ca^2+^ response in Lyn‐D3cpv cells on 0.6 kPa gel, but not in Kras‐D3cpv, suggesting that the local DRM‐specific Ca^2+^ enrichment is mainly via TRPM7 during cell–matrix adhesion (Figure S7, Supporting Information). Our finding hence suggests that TRPM7 is a crucial regulator of matrix rigidity‐dependent Ca^2+^ mobilization at DRM microdomains during the adhesion process. The colocalization of TRPM7 with Lyn, a DRM marker, as well as with paxillin and p‐FAK (Tyr397), provides additional supporting evidence that TRPM7 locally associated with FAK and integrin/focal adhesion complex (Figure S8, Supporting Information). As such, a putative connection between FAK and TRPM7 mediated by CSK‐actomyosin may contribute to the overall control of Ca^2+^ mobilization.^42^

## 3 Conclusions

**Figure 5:**
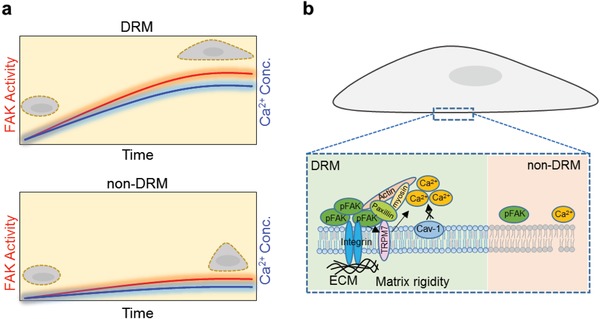
A proposed model of matrix rigidity‐dependent Ca^2+^ signals regulated by FAK during adhesion process. a) Ca^2+^ and FAK signaling is currently activated at DRM regions during the cell–matrix adhesion process, while they are less activated at non‐DRM. b) Matrix rigidity‐dependent Ca^2+^ mobilization at DRM is regulated by the functional support of the TRPM7 channel, but not Caveolin‐1. Integrin‐FAK complex could form interaction with TRPM7 via cytoskeleton (CSK)‐actomyosin network to regulate Ca^2+^ mobilization at DRM during cell–matrix adhesion process.

Previous evidence has shown that substrate stiffness directs hMSCs differentiation but with limited understanding on the underlying molecular mechanisms.^5^ Our results reveal differential Ca^2+^ and FAK signaling specifically occurring within DRM microdomains in hMSCs on hard and soft matrix at the initial stage of cell–matrix adhesion (**Figure** **5**a). As the adhesion process establishes the link between ECM and cytoskeleton, these initial differences can have profound effects on subsequent cellular fate. Such a study may shed light on the impact of the segregation of membrane microdomains on cellular responses to mechanical environment and on consequent functional outcomes.

In conclusion, we have shown that Ca^2+^ signals at DRM regions are regulated by FAK signaling during the adhesion process, and that this phenomenon is dependent on extracellular matrix rigidity in hMSCs. Additionally, our results demonstrate that FAK regulates Ca^2+^ mobilization and that this can be controlled by the proper localization of cholesterol and the functional support of TRPM7 (Figure 5b). These data provide new insights into the underlying mechanisms that regulate Ca^2+^ and FAK signals, and the relationship between biochemical/mechanical factors and cellular differentiation in hMSCs.

## 4 Experimental Section

*Construction of DNA Plasmids*: The Lyn‐FAK biosensor was generated by insertion of a raft‐targeting motif (MGCIKSKRKDNLNDDE) originated from Lyn kinase to the N‐terminus of the cytosolic‐FAK biosensor. Kras‐FAK biosensor was also constructed by insertion of a non‐raft‐targeting motif: a prenylation substrate sequence from Kras (KKKKKKSKTKCVIM) to the C‐terminus of the cytosolic‐FAK biosensor.^20^, ^23^ The DNA encoding the FAK biosensors that contain ECFP‐YPet pair were subcloned with the BamHI/EcoRI sites in pRSetB for the protein purification from *Escherichia coli*, and in pcDNA3.1 plasmid for the expression in mammalian cells. As FAK mutants, the kinase‐dead FAK with its kinase domain mutated (FAK KD) and the N‐terminal tail (containing 1–400 amino acids) of FAK (FAK NT) were used in this study.^23^ The membrane‐targeting Ca^2+^ biosensors based on FRET were generated in the same manner. The plasmids Lyn‐D3cpv and Kras‐D3cpv were constructed by fusion of a raft‐targeting motif: the myristoylation and palmitoylation sequence from Lyn kinase (MGCIKSKRKDNLNDDGVDMKT) to the N‐terminus of the D3cpv and a non‐raft‐targeting motif (KKKKKKSKTKCVIM) to the C‐terminus of the D3cpv.^43^ A dominant negative Caveolin‐1 mutant (Cav1 S80E) was used to disrupt caveolar organization.^44^ The caveolin‐1 was amplified by PCR and inserted into pcDNA3.1 by BamHI and EcoRI sites. The primers are forward 5′‐GCGCGGATCCGCCACCATGTCTGGGGGCAAATACGTAG‐3′ and reverse 5′‐TCCGGAATTCTTATATTTCTTTCTGCAAGTTGATG‐3′. The Cav1 S80E mutant was generated by using QuikChange Site‐Directed Mutagenesis Kit (Stratagene, La Jolla, CA).

*Cell Culture and Chemicals*: hMSCs (Lonza Walkersvile, Inc., Walkersvile, MD) were purchased from Lonza and maintained in mesenchymal stem cell growth medium (MSCGM, PT‐3001, Lonza) containing 10% fetal bovine serum (FBS), 2 × 10^−3^ m l‐glutamine, 100 U mL^−1^ penicillin, and 100 µg mL^−1^ streptomycin in a humidified incubator of 95% O_2_ and 5% CO_2_ at 37 °C. The DNA plasmids were transfected into the cells (transfection efficiency, 29.8%) by using Lipofectamine 2000 or LTX (Invitrogen, Carlsbad, CA) reagent according to the product instructions. PF228, ML‐7, methyl‐beta‐cyclodextrin (MβCD), and naltriben methanesulfonate hydrate were purchased from Sigma Aldrich (St. Louis, MO).

*RNA Interference Assays*: Small interfering RNA (siRNA) sequences targeting human TRPM7 (ON‐TARGETplus SMARTpool siRNA) and nontargeting control sequences were designed by Dharmacon RNAi Technology (Dharmacon Inc., Lafayette, CO). Cotransfection of 1–2 µg siRNA specific for TRPM7 (L‐005393‐00) or a nontargeting pool (D‐001810‐10‐05) along with the plasmid DNA was conducted according to the product instructions.

*Bis‐acrylamide‐PA Gel Fabrication*: The fabrication of PA hydrogel with a defined modulus of elasticity (*E* or stiffness), a characteristic of the ECM can be a useful technique to study the interactions of cells with their mechanical microenvironment. Such matrix substrate from PA gels can be created by simply changing relative concentration of acrylamide and bis‐acrylamide.^45^ PA gels were cast on amino‐silanized glass coverslips. 40% w/v acrylamide and 2% w/v bis‐acrylamide stock solutions (Bio‐Rad) were mixed to prepare PA solution and then the gel's stiffness was achieved by varying the final concentrations of PA solution (3 and 7.5%) and bis‐acrylamide cross‐linker (0.06 and 0.4%) for the corresponding stiffness of 0.6 (soft gel) and 40 kPa (hard gel). To polymerize the solutions, 2.5 µL of 10% w/v ammonium persulfate (APS; Bio‐Rad) and 0.25 µL of N,N,N9,N9‐tetramethylethylenediamine (TEMED; Bio‐Rad) were added to yield a final volume of 500 µL PA solution. To crosslink extracellular matrix molecules onto the gel surface, a photoactive cross‐linker, sulfo‐SANPAH (0.5 mg mL^−1^, sulfosuccinimidyl 6(4′‐azide‐2′‐nitrophenyl‐amino) hexanoate, Pierce) was used. For adhesion via integrins, 200 µL of a 0.1 mg mL^−1^ fibronectin solution (from bovine plasma, Sigma) was incubated overnight with the PA gel at 37 °C.

*Image Acquisition and Microscopy*: FRET sensor‐transfected cells were incubated for 1 h on 1% agarose dishes in a humidified incubator of 95% O_2_ and 5% CO_2_ at 37 °C to maintain in a state of suspension after detachment with 4 × 10^−3^ m EDTA in phosphate buffered saline (PBS). During imaging process, the cells were maintained with MSCGM in a chamber, which was designed to provide the constant humidified air containing 5% CO_2_, 10% O_2_, and 85% N_2_. The 37 °C degree of temperature throughout the samples in the chamber was maintained by a controlled heater (Nevtek ASI 400). Nikon Eclipse Ti inverted microscope with a cooled charge‐coupled device (CCD) camera was used for the image acquisition under perfect focus system (PFS) which allows for minimizing any focal change during cell–matrix adhesion. The pixel‐by‐pixel ratio images of FRET/ECFP were quantified after background subtraction in fluorescence intensity images of FRET and ECFP. The emission ratio images were computed and quantified by the MetaFluor software, and presented in the intensity modified display (IMD) mode. The following filter sets were utilized in the imaging experiments: dichroic mirror (450 nm), excitation filter for ECFP (420/20 nm), emission filter for ECFP (480/40), and FRET emission filter (535/25 nm).

*Immunofluorescence (IF) Staining and Confocal Microscopy*: IF staining was carried out on hMSCs seeded on 40 kPa substrate. Fixation was performed with 4% paraformaldehyde in PBS for 10 min at room temperature, followed by washing three times with PBS. Fixed cells were permeabilized with 0.1% Triton‐X100 (Sigma, Cat. No. STBG3972V) in PBS for 15 min and blocked with 3% BSA (bovine serum albumin) in PBS for 1 h at room temperature. The cells were then stained with primary antibodies, mouse anti‐TRPM7 (1:100, GeneTex, GTX41997), and rabbit anti‐phospho‐FAK (Tyr397; 1:200, ThermoFisher, Cat. No. 700255) overnight at 4 °C and secondary antibodies, FITC‐conjugated anti‐mouse IgG (1:100, Santa Cruz, sc‐516140) and CFL 555 conjugated anti‐rabbit IgG (1:200, Santa Cruz, sc‐516249) at room temperature for 1 h. After washing three times with PBS, the samples were mounted in VECTASHIELD medium containing DAPI (VECTOR Laboratories, H‐1200) and stored at 4 °C in the dark. Confocal images were acquired using a Leica laser scanning confocal microscope (TCS‐SP8) with a 40× Plan‐Apo objective**/**numerical aperture (NA) 1.40.

*Statistical Analysis*: All statistical data were expressed as the mean ± standard error of the mean (s.e.m.). Statistical evaluation was performed by unpaired *t*‐test and one‐way ANOVA using Graphpad Prism 6.0 software to determine the statistical differences between groups. A significant difference was determined by the *P*‐value (<0.05).

## Conflict of Interest

Y.W. is a scientific co‐founder of Cell E&G Inc. D.‐H.K is a co‐founder and scientific board member of NanoSurface Biomedical Inc. However, these financial interests do not affect the design, conduct, or reporting of this research.

## Supporting information

Supplementary
