# Supplementary material for: Matrix Rigidity‐Dependent Regulation of Ca2+ at Plasma Membrane Microdomains by FAK Visualized by Fluorescence Resonance Energy Transfer
Source: Adv Sci (Weinh). 2018 Dec 18;6(4):1801290. doi: 10.1002/advs.201801290 (PMC6382294; doi:10.1002/advs.201801290)
Supplement: Supplementary file 1 — Supplementary [file ADVS-6-1801290-s001.pdf]

Copyright WILEY-VCH Verlag GmbH & Co. KGaA, 69469 Weinheim, Germany, 2018.

ADVANCED SCIENCE
Open Access

Supporting Information

for *Adv. Sci.,* DOI: 10.1002/advs.201801290

Matrix Rigidity-Dependent Regulation of Ca^{2+} at Plasma Membrane Microdomains by FAK Visualized by Fluorescence Resonance Energy Transfer

Tae-Jin Kim, Lei Lei, Jihye Seong, Jung-Soo Suh, Yoon-Kwan Jang, Sang Hoon Jung, Jie Sun,* Deok-Ho Kim,* and Yingxiao Wang**

Supporting Information

Matrix rigidity-dependent regulation of Ca^{2+} at plasma membrane microdomains by FAK visualized by fluorescence resonance energy transfer

Tae-Jin Kim*, Lei Lei, Jihye Seong, Jung-Soo Suh, Yoon-Kwan Jang, Sang-Hoon Jung, Jie Sun*, Deok-Ho Kim*, and Yingxiao Wang*

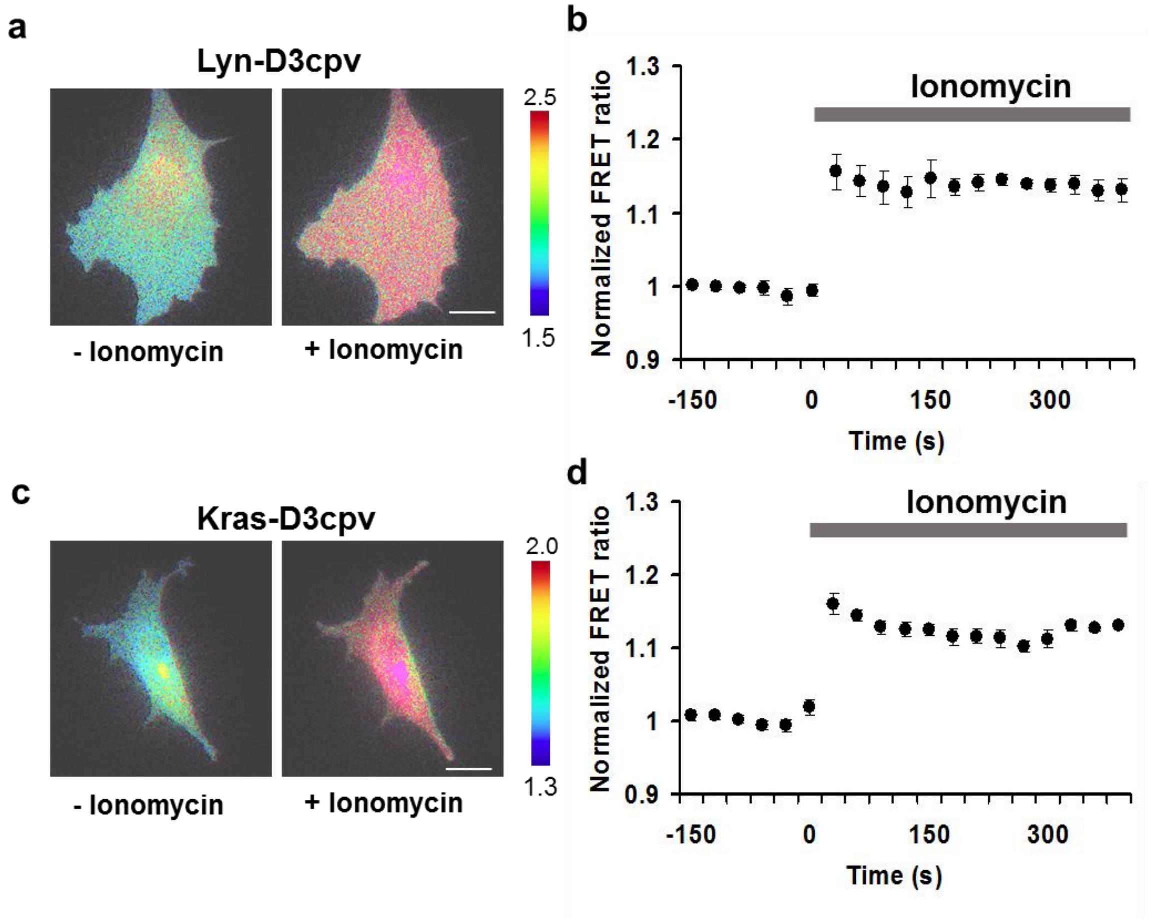

Figure S1. Capacity of Ca^{2+} FRET biosensors to target two different membrane microdomains. (a-d) Lyn-D3cpv and Kras-D3cpv show identical capacity of sensing in response to ionomycin. FRET images and ratio time courses represent the behavior of these two biosensors (n=3). Scale bar = 20 μm.

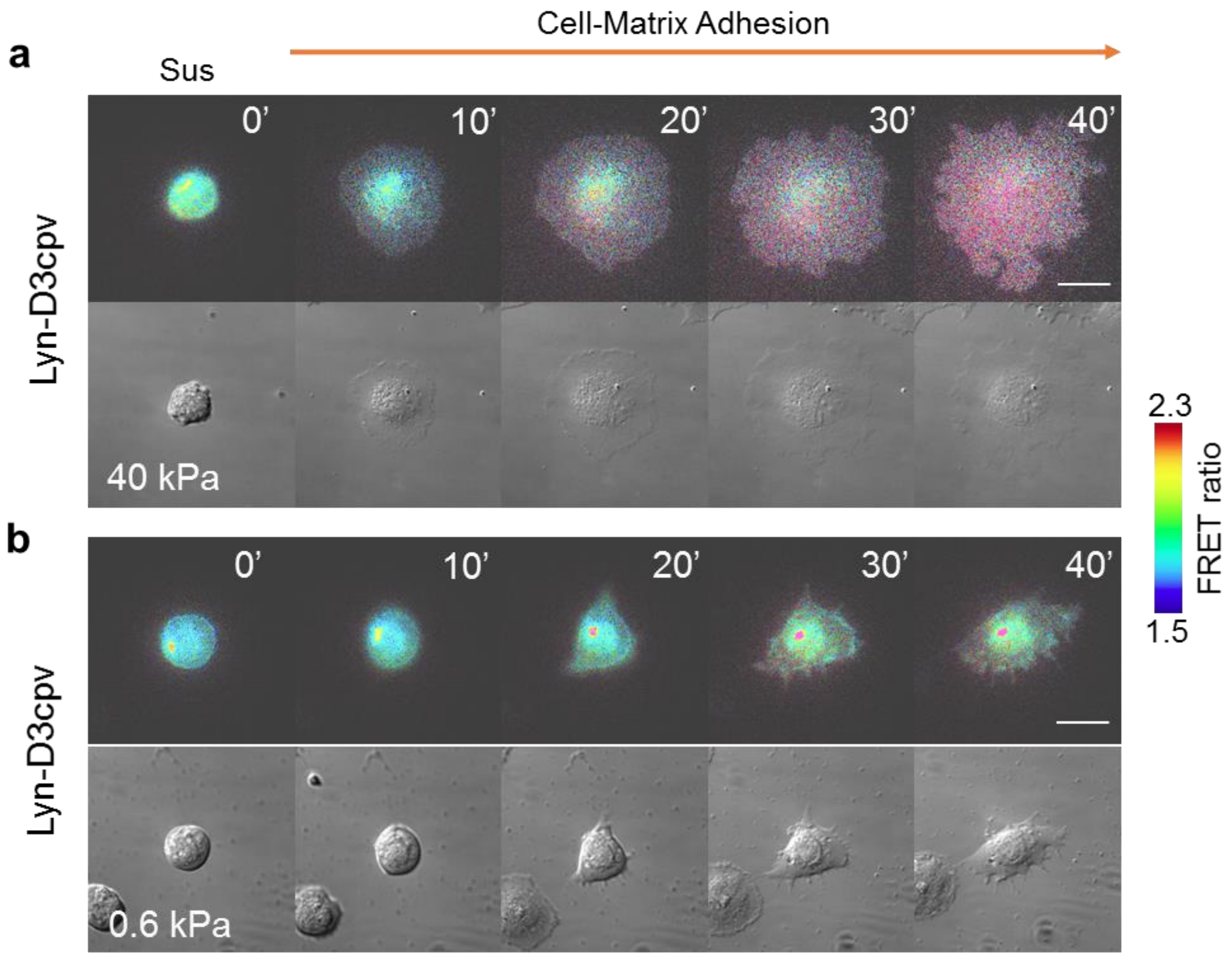

Figure S2. (a-b) FRET-based Ca^{2+} imaging targeting DRM regions in hMSCs seeded on to 40 kPa and 0.6 kPa gels. The hot and cold colors represent high and low FRET ratios indicating high and low Ca^{2+} activities, respectively. Scale bar = 20 µm.

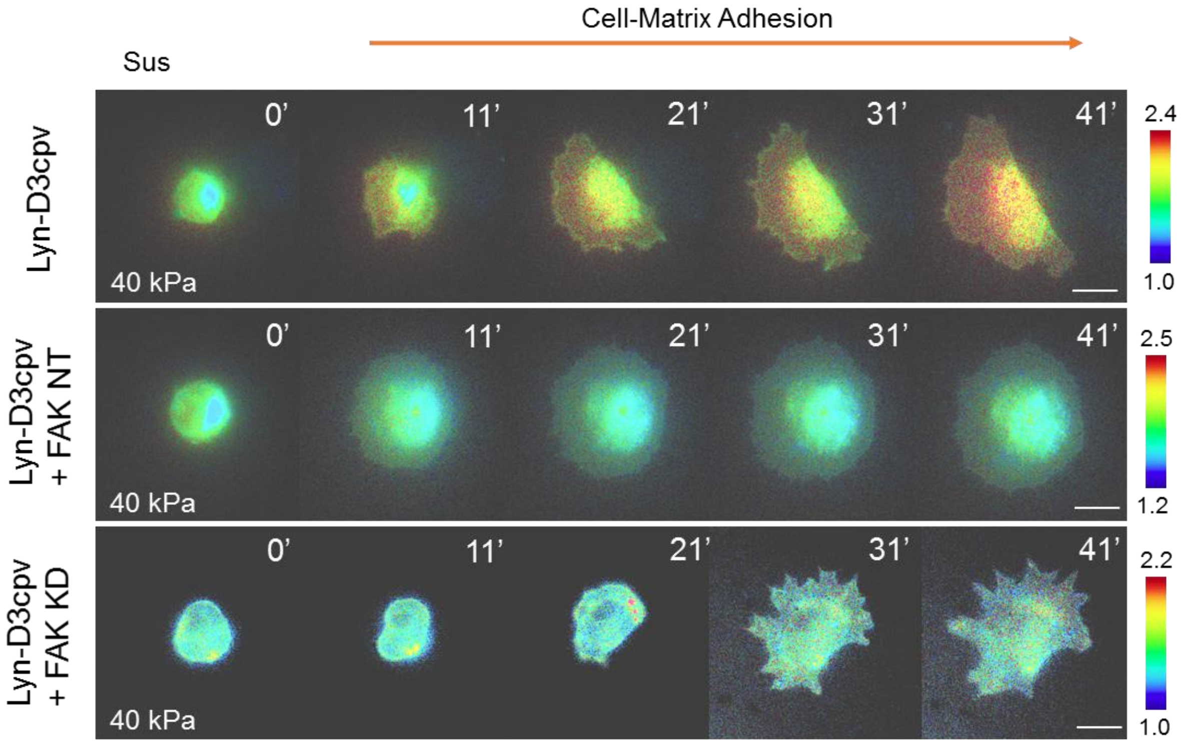

Figure S3. FRET images of the Ca^{2+} signal targeting DRM in hMSCs during the cell-matrix adhesion process in the absence or presence of FAK mutants. Scale bar = 20 µm.

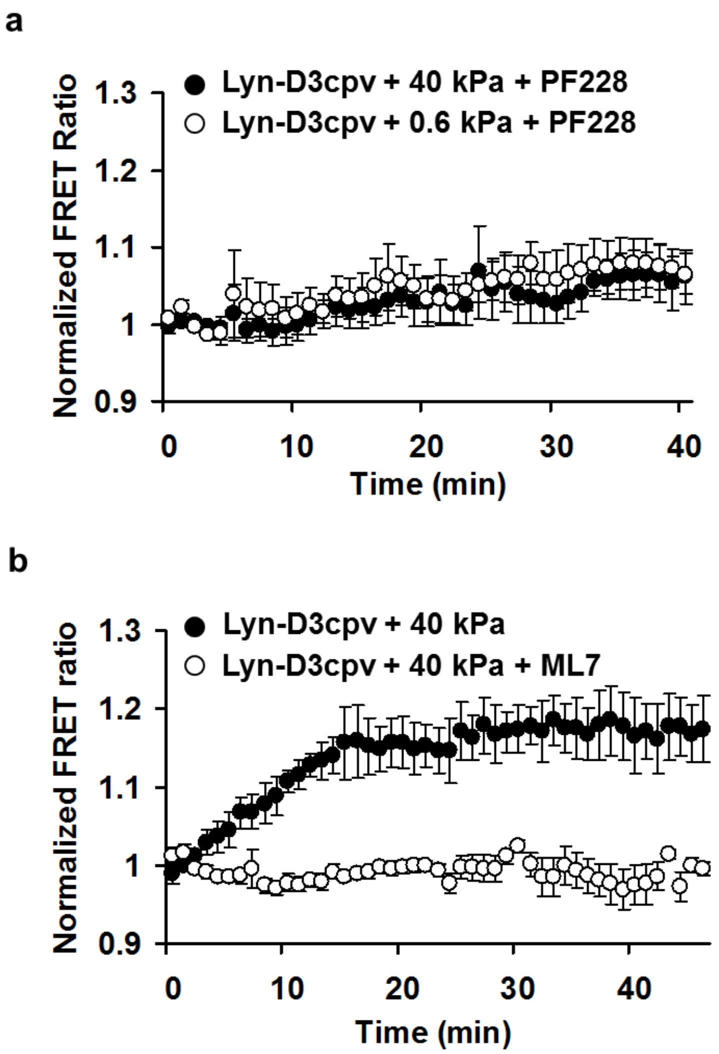

Figure S4. Changes in the FRET ratio of the Ca^{2+} signal at DRM when FAK or MLCK was inhibited. (a) The FRET ratio of the Ca^{2+} signal in hMSCs pretreated with PF228, a FAK inhibitor, before being seeded on 40 kPa or 0.6 kPa gels (n=4). (b) The FRET ratio of Ca^{2+} signal at DRM in hMSCs being seeded on 40 kPa gel in the absence or presence of ML-7, an inhibitor of MLCK (n=4-7).

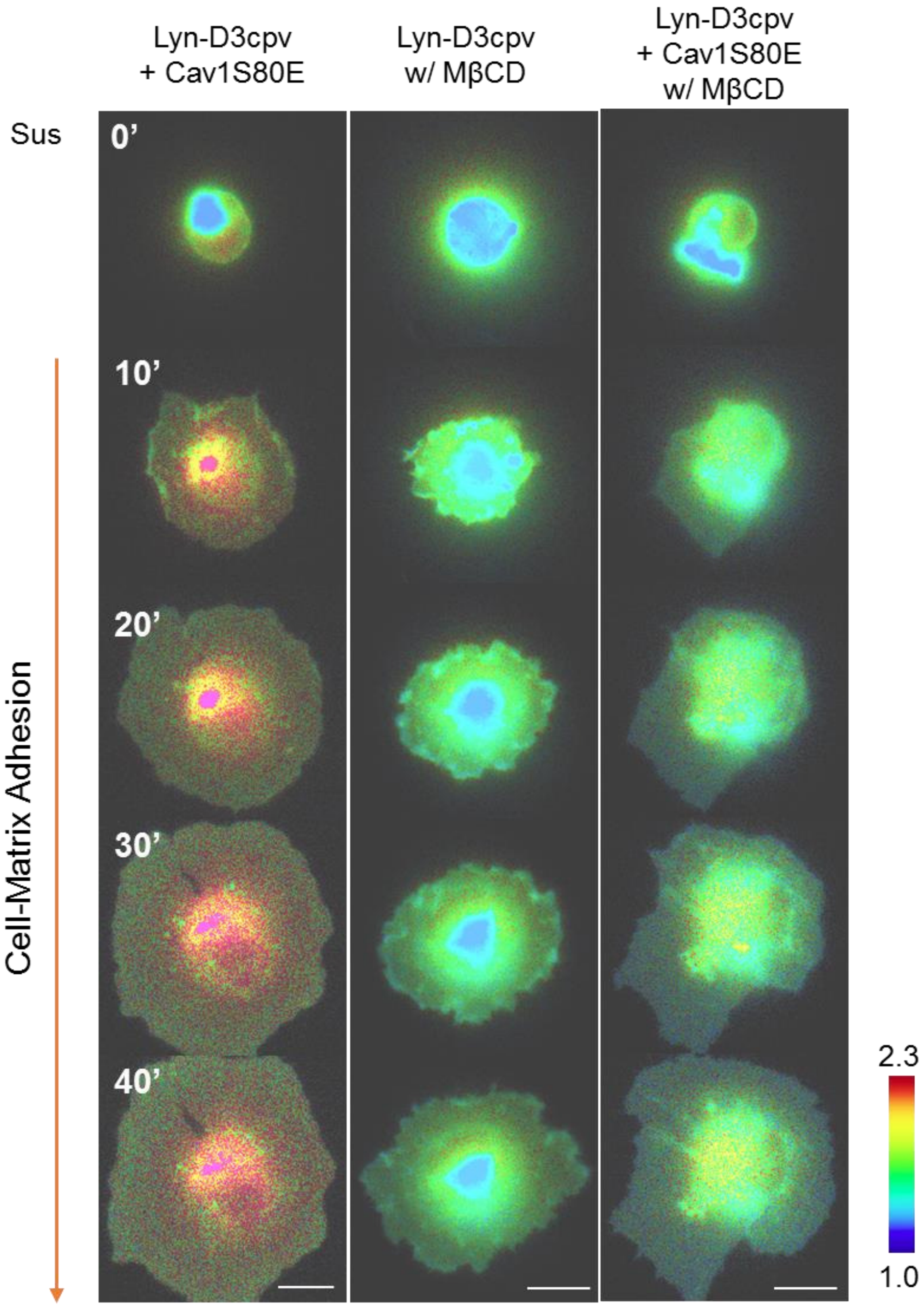

Figure S5. The FRET images of the Ca^{2+} signal at DRM in hMSCs during cell-matrix adhesion process in the presence of caveolin-1 mutant (Cav1S80E) and/or MβCD. Scale bar = 20 µm.

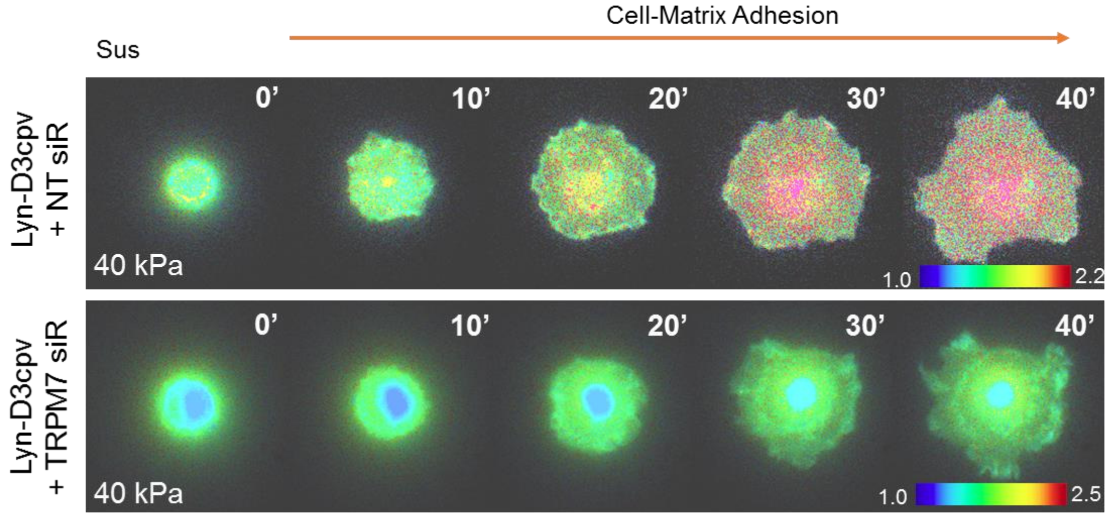

Figure S6. The FRET images of the Ca^{2+} signal at DRM in hMSCs during the cell-matrix adhesion process in the presence of TRPM7 siRNA or NT siRNA. Scale bar = 20 µm.

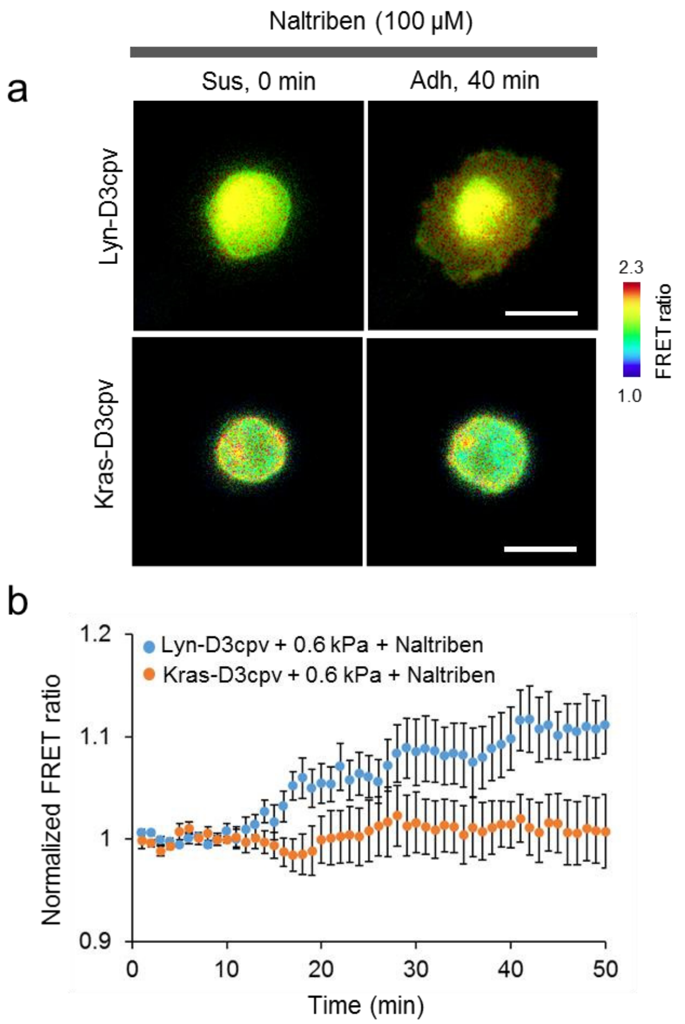

Figure S7. The effect of TRPM7 activator, Naltriben (100 uM) on Ca^{2+} signal at DRM and non-DRM in hMSCs cultured on 0.6 kPa gel. (a) Representative FRET ratio images of Lyn-D3cpv and Kras-D3cpv transfected cells in the presence of natriben (100 µM) before and after cell-matrix adhesion on soft substrate. (b) The time courses of Lyn-D3cpv and Kras-D3cpv transfected hMSCs in the presence of natriben (n=6-7). Scale bar = 20 µm.

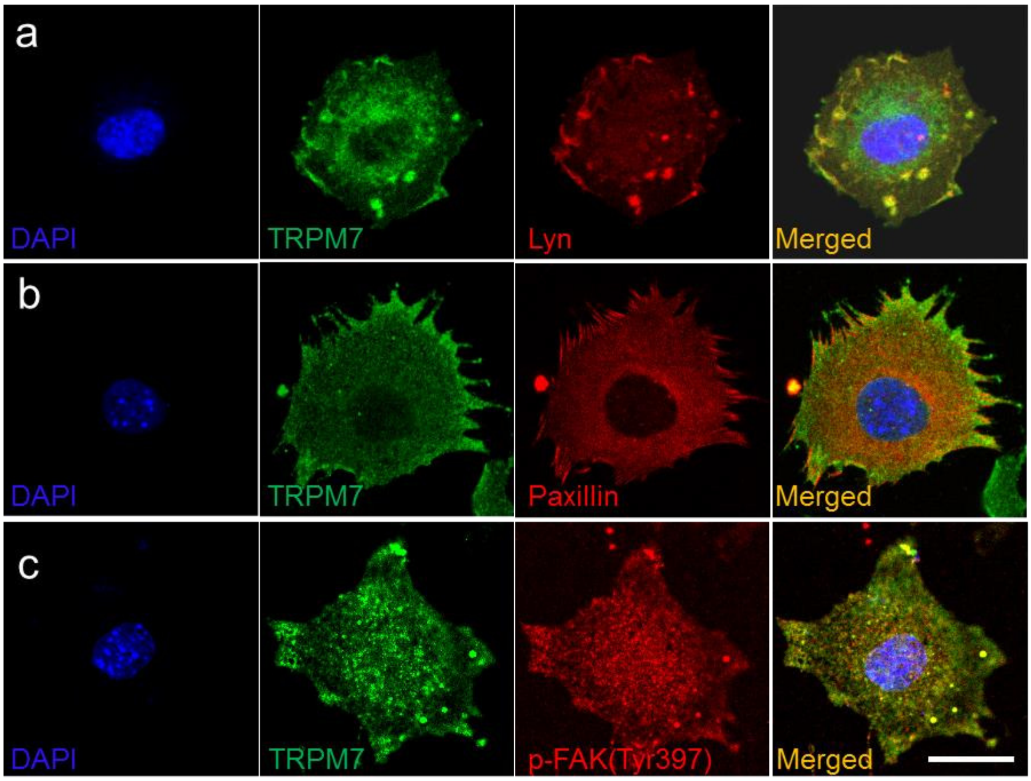

Figure S8. Colocalization of TRPM7 with Lyn tag fused with mCherry (a DRM specific marker), Paxillin and phospho-FAK (Tyr397). (a-c) hMSCs cultured on 40 kPa gel for 1hr were labeled with TRPM7 (green) and Lyn-mCherry (red) or Paxillin-mCherry (red) or p-FAK (red). DAPI (blue) was used to stain nucleus. Scale bar = 20 μm.
